# Estimating copy number using next-generation sequencing to determine ERBB2 amplification status

**DOI:** 10.1007/s12032-021-01482-1

**Published:** 2021-03-12

**Authors:** Kohei Nakamura, Eriko Aimono, Junna Oba, Hideyuki Hayashi, Shigeki Tanishima, Tetsu Hayashida, Tatsuyuki Chiyoda, Takeo Kosaka, Tomoyuki Hishida, Hirohumi Kawakubo, Minoru Kitago, Koji Okabayashi, Takeru Funakoshi, Hajime Okita, Sadakatsu Ikeda, Hiromasa Takaishi, Hiroshi Nishihara

**Affiliations:** 1 Genomics Unit, Keio Cancer Center, Keio University School of Medicine, 35 Shinanomachi, Shinjukuku, Tokyo 160-8582 Japan; 2 Department of Biomedical Informatics, Kansai Division, Mitsubishi Space Software Co., Ltd, Tokyo, Japan; 3 Department of Surgery, Keio University School of Medicine, 35 Shinanomachi, Shinjukuku, Tokyo 160-8582 Japan; 4 Department of Obstetrics and Gynecology, Keio University School of Medicine, 35 Shinanomachi, Shinjukuku, Tokyo 160-8582 Japan; 5 Department of Urology, Keio University School of Medicine, 35 Shinanomachi, Shinjukuku, Tokyo 160-8582 Japan; 6 Division of Thoracic Surgery, Department of Surgery, Keio University School of Medicine, 35 Shinanomachi, Shinjukuku, Tokyo 160-8582 Japan; 7 Department of Dermatology, Keio University School of Medicine, 35 Shinanomachi, Shinjukuku, Tokyo 160-8582 Japan; 8 Department of Diagnostic Pathology, Keio University School of Medicine, 35 Shinanomachi, Shinjukuku, Tokyo 160-8582 Japan; 9 Cancer Center, Tokyo Medical and Dental University, 1-5-45 Yushima, Bunkyo-ku, Tokyo, 110-8510 Japan; 10 Keio Cancer Center, Keio University School of Medicine, 35 Shinanomachi, Shinjukuku, Tokyo 160-8582 Japan; 11 Department of Obstetrics and Gynecology, Kumagaya General Hospital, Saitama, 360-8657 Japan

**Keywords:** Breast cancer, ERBB2, Gene copy number, Immunohistochemistry, Next-generation sequencing

## Abstract

Assessing Erb-b2 receptor tyrosine kinase 2 (*ERBB2*) amplification status in breast and gastric cancer is necessary for deciding the best therapeutic strategy. Immunohistochemistry (IHC) and fluorescence in situ hybridization (FISH) are currently used for assessing protein levels and gene copy number (CN), respectively. The use of next-generation sequencing (NGS) to measure *ERBB2* CN in breast cancer is approved by the United States Federal Drug Administration as a companion diagnostic. However, a CN of less than 8 is evaluated as “equivocal”, which means that some ERBB2 amplification cases diagnosed as “HER2 negative” might be false-negative cases. We reviewed the results of gene profiling targeting 160 cancer-related genes in breast (*N* = 90) and non-breast (*N* = 19) cancer tissue, and compared the *ERBB2* CN results with the IHC/FISH scores. We obtained an estimated CN from the measured CN by factoring in the histological proportion of tumor cells and found that an *ERBB2*-estimated CN of 3.2 or higher was concordant with the combined IHC/FISH outcome in 98.4% (88/90) of breast cancer cases, while this was not always evident among non-breast cancer cases. Therefore, NGS-estimated *ERBB2* CN could be considered a diagnostic test for anti-HER2 therapy after the completion of adequate prospective clinical trials.

**Supplementary Information:**

The online version contains supplementary material available at 10.1007/s12032-021-01482-1.

## Introduction

Human epidermal growth factor receptor 2 (HER2), the protein encoded by the Erb-b2 receptor tyrosine kinase 2 (*ERBB2*) gene, is one of the main therapeutic targets in human cancers [1]. HER2 is a transmembrane receptor tyrosine kinase that belongs to the HER family, and its overexpression leads to homodimerization and heterodimerization with other HER family-member proteins [2], triggering activation of the phosphoinositide-3-kinase/Akt and mitogen-activated protein kinase pathways [3], resulting in tumor proliferation, differentiation, apoptosis regulation, angiogenesis, and invasion [4].

HER2 overexpression is used as a biomarker in breast and gastric cancers to predict the response to anti-HER2 monoclonal antibody therapies (trastuzumab and pertuzumab) and small-molecule HER2 kinase inhibitors (lapatinib) [5, 6]. Clinical laboratories stain for HER2 protein by immunohistochemistry (IHC), or detect *ERBB2* amplification by FISH or chromogenic in situ hybridization. Amplification of *ERBB2* is a potential therapeutic target in other cancer types, including lung, bladder, endometrial, ovary, colorectal, esophageal, and bile duct cancers [7–14]. There are consensus scoring guidelines for these techniques for breast and gastric cancers [15, 16]; however, these have not been established for other cancer types.

Next-generation sequencing (NGS) is being increasingly adopted in clinical settings for the identification of multiple genomic changes, such as single-nucleotide variants and copy number (CN) changes. According to the current guidelines, hybrid capture-based NGS detection of *ERBB2* CN amplification (CN higher than 8) is approved as a companion diagnostic for breast and upper gastrointestinal carcinomas by the United States Federal Drug Administration. However, according to their guidelines, a slight degree of amplification (CN over 3 but lower than 8) is diagnosed as ‘equivocal’, and the diagnostic value of an NGS equivocal CN status for *ERBB2* has not yet been determined, as the accurate *ERBB2* CN therapeutic cutoff value has not been established. In the present study, we aimed to compare targeted NGS-based *ERBB2* CN estimation with HER2 IHC or FISH for breast and other cancer types, and to define the accurate *ERBB2* CN therapeutic cutoff value.

## Results

### Patients and clinicopathological features

The present study group comprised 90 patients with breast cancer and 19 patients with other cancer types, and their characteristics (procedures performed and sample type) are listed in Table 1. The details of their clinical and pathological features are summarized in Table S1 (breast cancer) and Table S2 (non-breast cancer). Among the 90 breast cancer samples, 13 were derived from core needle biopsies and 77 from surgical resections, and the age of the patients ranged from 37 to 81 years (median 55). Eighty-four percent of patients (*N* = 76) had invasive ductal carcinoma, followed by 11% (*N* = 10) with ductal carcinoma in situ. According to the International Union Against Cancer staging system 8th edition, 94.4% (*N* = 85) had Tis/T1/T2, while 5.6% (*N* = 5) had T3/T4. Regarding the estrogen receptor (ER), progesterone receptor (PgR), and Ki67 IHC status of the cohort, 49% (*N* = 44) had ER-positive tumors, while 40% (*N* = 36) had PgR-positive, and 50% (*N* = 45) had Ki67-positive tumors. The percentage of tumor cells was estimated independently by three pathologists on hematoxylin and eosin-stained slides, where the tumor area was marked (histological proportions of tumor cells in Tables S1 and S2).

**Table 1:** Sample types and procedures performed in 109 cancer patients recruited for the present study

Type of cancer		Total	Procedure	
			Core	Excision
Breast		90	13	77
Non-Breast		19	2	17
	Gastric cancer	3	0	3
	Lung cancer	2	0	2
	Colorectal cancer	2	0	2
	Bladder cancer	2	2	0
	Extra-mammary Paget's disease	2	0	2
	Renal pelvic cancer	1	0	1
	Cervical cancer	1	0	1
	Endometrial cancer	1	0	1
	Ovarian cancer	1	0	1
	Gallbladder cancer	1	0	1
	Prostate cancer	1	0	1
	Uterine sarcoma	1	0	1
	Anal fistula	1	0	1

### HER2 IHC, FISH, and NGS-based ERBB2 CN among patients with breast cancer

**Fig. 1:**
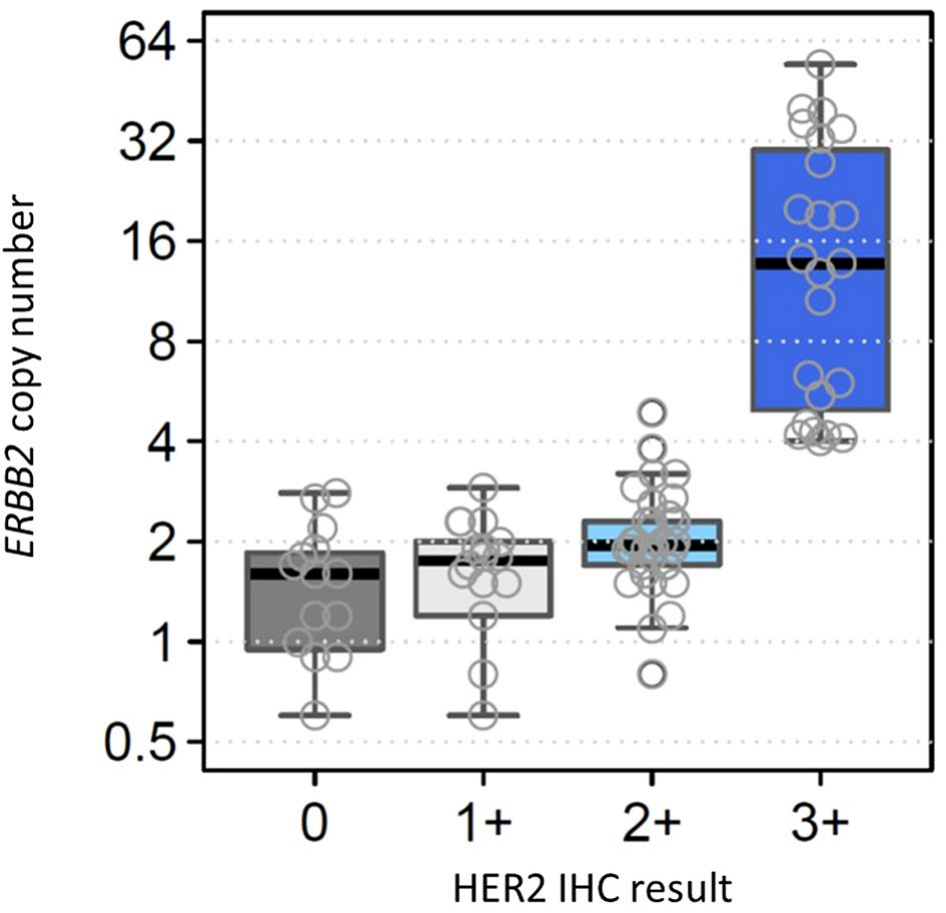
Estimated *ERBB2* CN determined by NGS and HER2 IHC results among breast cancer cases. Statistically significant differences were observed between IHC 0 group (*N* = 15) vs. IHC 2 + group (*N* = 34) (*p* = 0.04), IHC 0 group vs. IHC 3 + group (*N* = 23) (*p* = 1.5e-06), IHC 1 + group (*N* = 18) vs. IHC 3 + group (*p* = 3.2e-07), and IHC 2 + group vs. IHC 3 + group (*p* = 2.2e-09). Boxplots were generated in R using default settings. Each box spans the 25th to 75th percentile range of the data, i.e., the interquartile range (IQR), and the middle line represents the median value. Whiskers extend 1.5 times the IQR from the edge of the box. *ERBB2*, Erb-b2 receptor tyrosine kinase 2; NGS, next-generation sequencing; HER2, Human epidermal growth factor receptor 2; IHC, immunohistochemistry

The comparison between the estimated *ERBB2* CN determined by NGS and the HER2 IHC score is provided in Fig. 1. Statistically significant differences in estimated CN were found among different IHC groups. The comparison between the NGS-derived *ERBB2*-estimated CN and the HER2 IHC/FISH results is shown in Figure S1. Table S1 summarizes the HER2 IHC scoring, FISH results, *ERBB2* CN status and measured/estimated CN by NGS, and *ERBB2* mutation status in the breast cancer cohort. Among samples with amplified *ERBB2*, the estimated CN ranged from 3.2 to 54.3 (median 11.7). There was no significant difference in the proportion of tumor cells between the *ERBB2*-amplified and *ERBB2* neutral CN cases (data not shown). For HER2 IHC, 23 of 90 (26%) tumors showed 3 + staining, 34 (38%) showed 2+ , 18 (20%) showed 1+ , and 15 (17%) showed no staining. FISH was performed on 34 samples, all of which showed an HER2 IHC score of 2+ , and four of which had *ERBB2* amplification according to NGS. Notably, only 2 cases (5.8%) had positive results in FISH, and both had *ERBB2* amplification according to NGS. Of the 27 *ERBB2*-amplified cases determined by NGS, 23 tumors showed strong positive HER2 staining (IHC score 3+). The four remaining *ERBB2*-amplified cases displayed HER2 2+ staining, two of which also had a positive FISH result, but the other two had negative FISH results. Among the four cases that met the criteria for amplification, two had a negative FISH result; one had an estimated CN = 4.8 and IHC2+ /FISH − , whereas the remaining case had an estimated of CN = 3.1 and IHC2+ /FISH − . Of the 63 cases without *ERBB2* amplification according to NGS, 15 showed no HER2 IHC expression, and 48 had mild staining with an IHC score of 1+ and 2+ . Among the 30 cases with 2+ IHC staining and no *ERBB2* amplification, no case was positive for FISH.

### HER2 IHC, FISH, and ERBB2 CN among patients with non-breast cancer with ERBB2 amplification

Table S2 summarizes the HER2 IHC scoring, *ERBB2* CN status, measured/estimated CN by NGS, and *ERBB2* mutation status in non-breast cancer cases in the present study. Among 19 patients with *ERBB2* amplification and an estimated CN of more than 3.0 by NGS, only 12 cases had HER2 IHC 3+ , four cases had 2+ , one case had 1+ , and two cases had no staining. The comparison between *ERBB2* estimated CN and HER2 IHC results is provided in Figure S2. As a HER2 IHC scoring system has not been established for cancers other than breast and gastric cancers, we sought to evaluate HER2 protein expression by IHC in solid cancers using the breast cancer scoring system, and in luminal cancers using the gastric cancer scoring system. The IHC findings in all non-breast cancer cases are shown in Figure S3. All 19 non-breast tumors had *ERBB2* amplification (estimated CN above 3.0) and varying HER2 IHC scores (Table S2).

### Determining thresholds of estimated CN for NGS-based ERBB2 amplification among cases with breast cancer

**Fig. 2:**
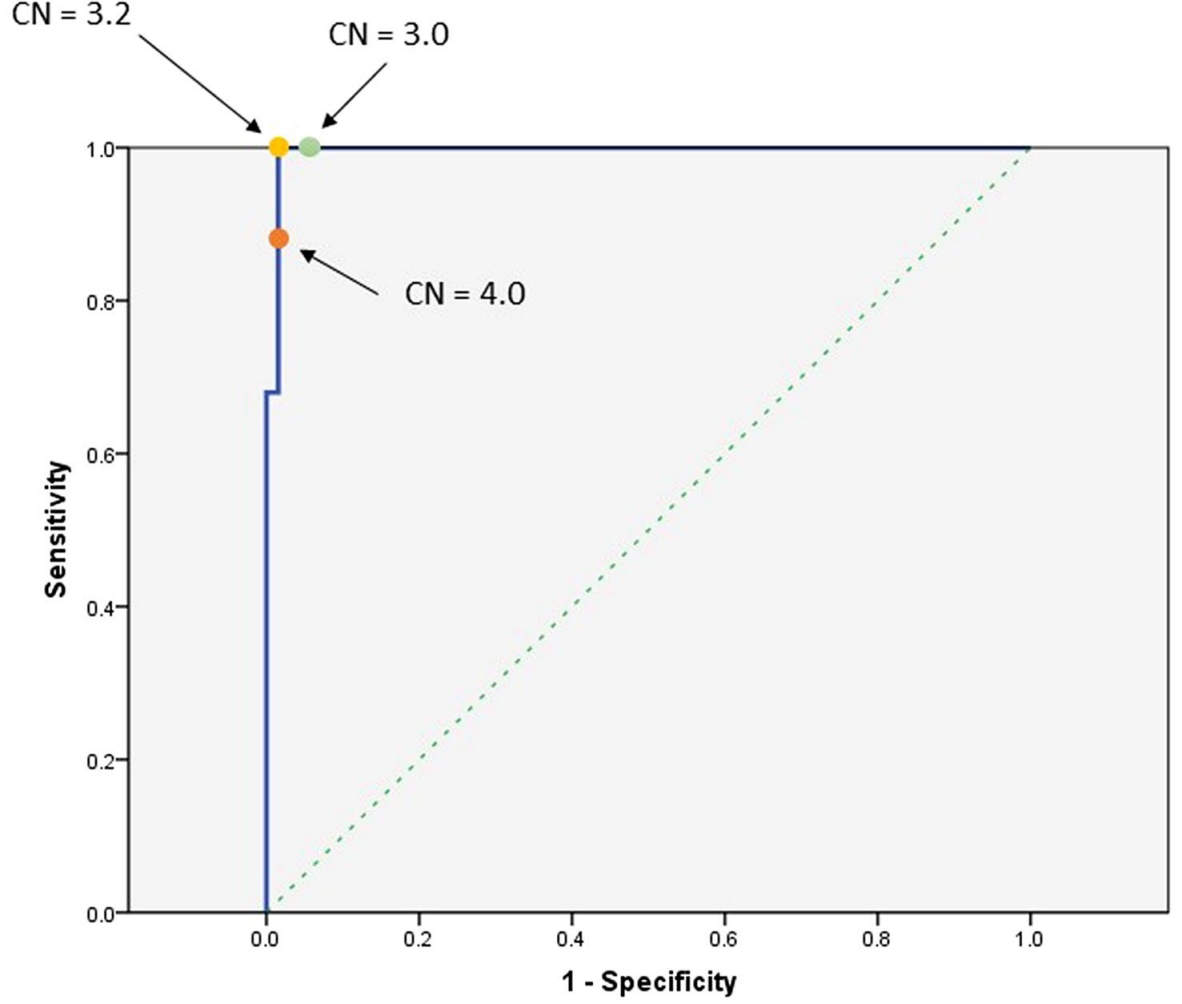
Receiver operating characteristics curve for different CN threshold values. For lower threshold values, the pipeline calls everything as amplified, which are all false positives (x axis); for higher threshold cutoff values, our ability to correctly call real events decreases. A threshold of 3.2 has the best sensitivity and specificity

**Fig. 3:**
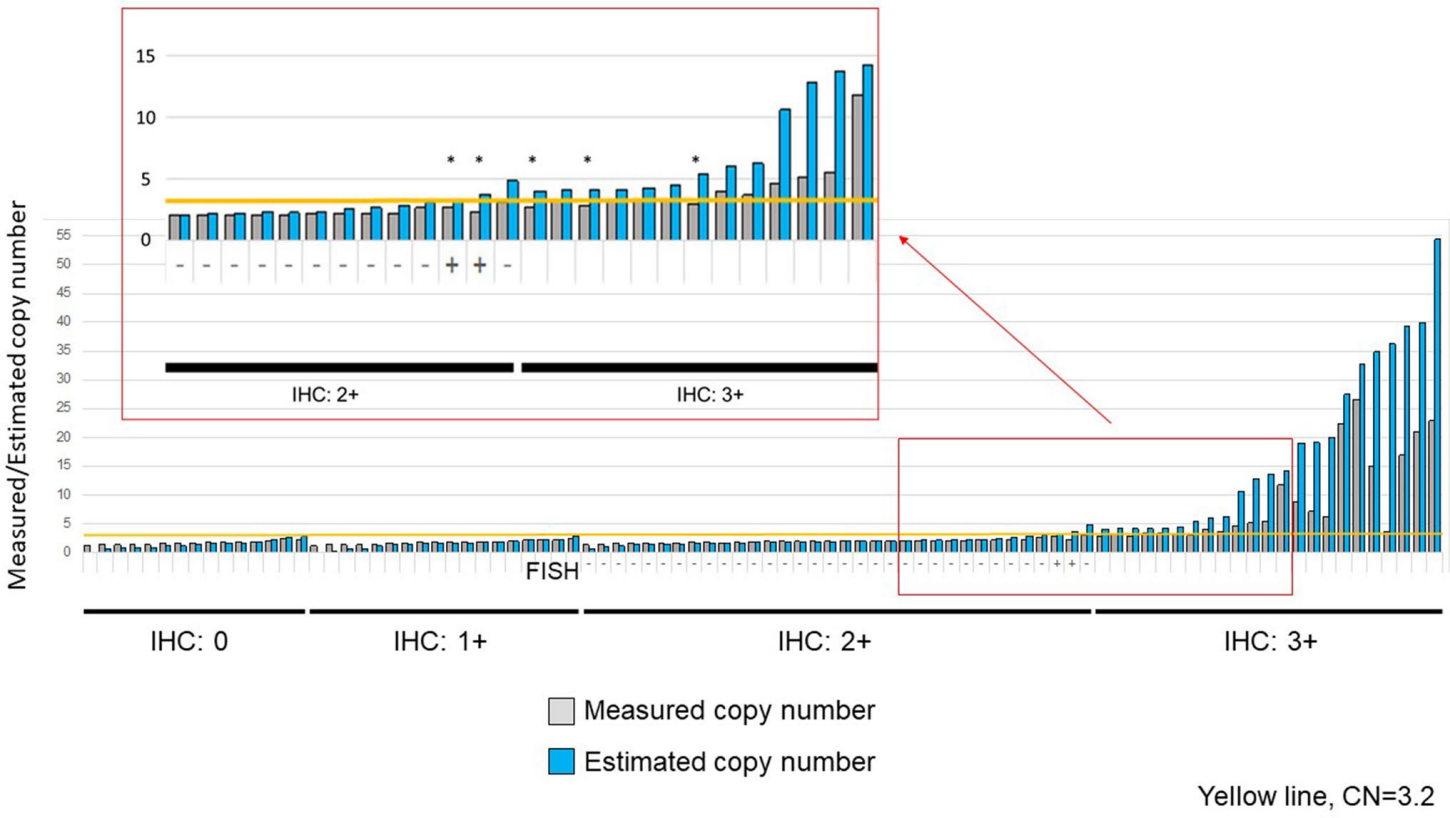
*ERBB2* measured/estimated CN and HER2 IHC/FISH results. Yellow line is CN = 3.2. Five patients marked with an asterisk had estimated CN of over 3.2 but measured CN under 3.2. Erb-b2 receptor tyrosine kinase 2; HER2, Human epidermal growth factor receptor 2; IHC, immunohistochemistry; FISH, fluorescence in situ hybridization

Current breast cancer treatment guidelines indicate that patients with either HER2 IHC3 + or HER2 IHC2 + /FISH + should be considered for anti-HER2 therapies. We evaluated the association between NGS-derived *ERBB2* estimated CN and the IHC/FISH results. All cases with HER2 IHC3 + or IHC2 + /FISH + had *ERBB2* amplification with an estimated CN of more than 3.2. The receiver operating characteristic (ROC) curve (Fig. 2) shows the relationship between sensitivity and specificity for every possible cutoff of the *ERBB2* estimated CN and the HER2 IHC 3 + and IHC 2 + /FISH + outcomes. A cutoff of estimated CN = 3.2 achieved the maximum performance with 100% sensitivity and 98.5% specificity for an IHC 3 + and IHC 2 + /FISH + outcome in our breast cancer cohort. A cutoff value of estimated CN = 3.0 also achieved high sensitivity and specificity (100% and 97%, respectively), while a cutoff value of CN = 4.0 had decreased sensitivity of 88% and 98.5% specificity. Therefore, we defined *ERBB2* estimated CN = 3.2 as the cutoff for therapeutic recommendation. The relationship between the NGS-derived *ERBB2* measured/estimated CN and HER2 IHC and FISH results is shown in Fig. 3. Samples from the HER2 IHC 3 + group tended to have a greater discordance between measured and estimated CNs. There were five samples with measured CNs less than 3.2, while their estimated CNs were more than 3.2.

### Genomic alterations in patients with breast cancer

Tumors with a neutral CN for *ERBB2* included three cases with *ERBB2* mutations. The pathogenicity of all these variants was determined to be of unknown significance by our mutation calling algorithm.

## Discussion

With the advent of personalized cancer therapies, it is important to determine the expression levels of various molecular markers simultaneously in order to design effective therapeutic strategies. NGS has this capacity, as it can provide results with only a limited amount of sample material. However, clear diagnostic guidelines are necessary for the clinical use of NGS. We determined an *ERBB2* estimated CN threshold of 3.2 for recommending anti-HER2 therapy in breast cancer cases. A cutoff value of estimated CN = 3.0 also achieved high concordance with IHC3+ , indicating that only hemiallelic 2 × amplification causes IHC3 +status. This threshold (estimated CN = 3.2) was capable of identifying two additional cases that had been omitted by conventional methods, suggesting that there is heterogeneity in FISH sensitivity (Figure S4), with only 50% of cases with *ERBB2* amplification being FISH positive. Follow-up studies on the discordance between FISH and NGS amplification results are required to determine whether anti-HER therapy should be recommended for cases with NGS-derived amplification and IHC2+ /FISH − results. Although these cases did not meet the criteria by conventional IHC/FISH methods, they did satisfy the accepted amplification threshold using the NGS method. These cases highlight the promising capacity of NGS to offer therapeutic recommendations that could not have been possible with the IHC/FISH criteria. It is essential to observe the therapeutic response to the genotype-matched therapy in these equivocal cases, as well as to gather and observe similar cases to ascertain the validity of this new method.

All 19 non-breast tumors showed *ERBB2* amplification (estimated CN above 3.0) but their HER2 IHC score varied, indicating that NGS-derived CN amplification and IHC scoring systems are not as concordant in non-breast cancers as in breast cancers. If we apply the breast or gastric IHC scoring system to our non-breast/non-gastric tumor samples, an *ERBB2* estimated CN gain of 8 or more (estimated CN ≥ 8) would be equivalent to an IHC3 + score (90% sensitivity and 75% specificity). However, it is possible that establishing the cutoff point as high as 8 (instead of 3.2) would lead to the omission of patients who might have a favorable response to anti-HER2 treatment. Evaluating a large number of non-breast tumors for HER2 protein expression, including tumors without *ERBB2* amplification, is necessary to examine the potential clinical use of NGS-based *ERBB2* amplification status relative to the HER2 IHC score, as well as to determine whether a high estimated CN value threshold should be considered for non-breast cancers. Furthermore, given the behaviors of these marker in different tumor contexts, it is recommended that each tumor type should be analyzed separately.

Estimated CN gain detection is impacted by the proportion of tumor cells, with low or borderline amplification being most affected. Therefore, we estimated the true CN in NGS using the measured CN and the proportion of tumor cells. If the cutoff estimated CN = 3.2 is applied to the measured CN as a diagnostic tool, then these five patients would not be eligible for anti-HER therapies, and the sensitivity would fall to 80%. As the estimated CN takes the proportion of tumor cells into account, it is a better reflection of the actual tumor context. Accordingly, the estimated CN should be used for daily clinical reporting and evaluation of CN status. NGS is based on amplicon sequencing; therefore, exact CN estimation can be achieved with a relatively low sequencing depth. In the present study, we did not use matched normal blood samples, only tumor samples. As such, the estimated CN is not influenced by the germline CN. A previous study used a log2 fold change of 1.5 as the cutoff to detect *ERBB2* amplification, achieving 95% sensitivity and 100% specificity [17]. However, they used peripheral blood as the normal control and estimated the CN by comparing the CN of the tumor sample with that of the peripheral blood, without compensating for the tumor content. Therefore, especially in cases with low tumor content, the estimated CN deviated greatly from the actual CN. Furthermore, if the germline CN is lost or amplified, the estimated CN also deviates from the actual CN. Comparably, our CN cutoff is not affected by these factors, and is less likely to result in costly errors in clinical decision making. Finally, NGS also overcomes the inter- and intra-observer variabilities.

The small size of biopsy specimens may lead to false-negative results. Thus, variability in the histological features of cancers may be associated with established molecular intratumoral heterogeneity and variations in treatment response [6, 7]. In some samples, there is only a small amount of the specimen available for molecular testing, which may not be representative of the whole tumor. In cases with discrepancies between IHC and NGS results, reflex testing on the same specimen by FISH would be recommended if there is sufficient material remaining, or an alternate tumor block. This information is useful for judging *ERBB2* amplification to improve precision medicine.

There were two main limitations to the present study. First, for non-breast cancers, we only evaluated tumors with an estimated CN of 3.0 or higher. Therefore, we could not draw any conclusion or inference about the possible relationship between *ERBB2* estimated CN and IHC score. Second, our study did not investigate the response to anti-HER2 treatment based on *ERBB2* amplification using our criteria because of the short follow-up period between the NGS testing and the manuscript preparation. In future studies we plan to evaluate the treatment response in cases using our criteria for therapeutic recommendations. Turnaround time is an additional consideration when using NGS alone to determine *ERBB2* estimated CN. Comprehensive NGS assays, which yield precise CN results, are not fast, with a turnaround time of at least 14 working days. While this is longer than that expected for IHC or FISH alone, the overall time can be comparable if assays are performed sequentially, accounting for the time required for collection and transportation of material.

In conclusion, the present study showed that NGS can provide accurate *ERBB2* CN status, enabling simultaneous testing for other potentially actionable genomic alterations. Our *ERBB2* estimated CN cutoff for therapeutic recommendation is an acceptable solution when both the *ERBB2* status and comprehensive molecular profiling are required. We believe that our cutoff value for the *ERBB2* estimated CN determined by NGS can be added to the breast cancer treatment guidelines regarding anti-HER2 therapies. Although estimated CN alone could potentially be used without IHC or FISH for cases with markedly high estimated CN, confirming the results with IHC or FISH would strengthen the finding, especially when the estimated CN is marginal (around 3). An NGS approach may also be applicable to various other types of cancer for which an IHC/FISH scoring system has not been established. This method will be especially useful when biological material is limited in amount, but screening of targetable gene aberrations is needed for alternative therapeutic options. Despite the rise in NGS technology use throughout diagnostic laboratories, inherent variations among platforms, assay design, and data analysis indicate the need to update HER2 guidelines by adding criteria for NGS-based evaluation of ERBB2 status.

## Materials and methods

### Patients

This retrospective study was approved by the ethics committee of the Keio University (approval number: 20080015) and was conducted in accordance with the Declaration of Helsinki and Title 45, U.S. Code of Federal Regulations, Part 46, Protection of Human Subjects, effective December 13, 2001. All patients provided written informed consent. We enrolled 90 patients with breast cancer with/without *ERBB2* amplification, and 19 patients with other cancer types with *ERBB2* amplification (estimated CN of more than 3.0) (Table 1), who underwent surgery at Keio University. Resected specimens were used for the NGS assay. For breast cancer specimens with low histological proportion of tumor cells, we used the specimens from the core needle biopsy performed before surgery.

### NGS

Genomic testing was performed in-house using the PleSSision testing platform (Keio University Hospital, Tokyo, Japan). Briefly, genomic DNA was extracted from 10-µm-thick formalin-fixed paraffin-embedded (FFPE) tissue sections of tumor specimens using the Maxwell RSC FFPE Plus DNA Kit (Cat.AS1720, Promega, Madison, USA) according to the manufacturer’s instructions. DNA quality was checked by calculating the DNA integrity number (DIN), using an Agilent 4200 TapeStation (Agilent Technologies, Waldbronn, Germany); all analytes had DIN ≥ 2.0. Libraries were generated from 80 (DIN ≤ 2.5) or 160 (DIN > 2.5) ng of DNA per sample using the Human Comprehensive Cancer Panel, GeneRead DNAseq Panel PCR kit V2, GeneRead DNA Library I Core Kit, and GeneRead DNA Library I Amp Kit (Qiagen, Hilden, Germany) and the library quality was assessed using the Agilent D1000 ScreenTape (Agilent Technologies). Targeted amplicon exome sequencing was performed using a 160 cancer-related gene panel as previously described [18, 19]. The targeted regions of all 160 genes were specifically enriched using oligonucleotide probes. The enriched libraries were sequenced with a paired-end (150 bp × 2) sequencing method using the NextSeq sequencing platform (Illumina, San Diego, CA, USA), resulting in a mean depth of 500. The sequencing data were analyzed using the GenomeJack bioinformatics pipeline (Mitsubishi Space Software Co., Ltd., Tokyo, Japan) (http://genomejack.net/) as previously described [20]. The proportion of tumor cells ranged from 5 to 80% (median 45%). The estimated CN of the tumor cells was calculated by the following formula: estimated CN = (measured CN − 2)/proportion of tumor cells + 2.

### IHC

IHC for HER2 was performed on 4-µm thick FFPE whole-tissue sections using the PATHWAY anti-HER-2/neu rabbit monoclonal antibody (clone 4B5, Ventana Medical Systems, Tokyo, Japan) on the Leica BOND-III (Leica Microsystems, Wetzlar, Germany). External positive controls were tested with each run. To determine the level of HER2 expression, the membrane staining pattern was estimated and scored on a scale of 0 to 3 + for breast^15^ and gastric^16^ cancers. An established breast cancer HER2 IHC scoring method [15] was applied to other solid cancers including lung, bladder, renal pelvic, cervical, endometrial, ovary, and prostate cancers, uterine sarcoma, and extra-mammary Paget’s disease; for gastric cancers and cancers with clear luminal structure (colorectal, gallbladder, and fistula cancers), the gastric cancer scoring system [16] was used. Scoring was performed independently by three pathologists. The IHC for the ER and PgR was performed using the CONFIRM rabbit monoclonal antibodies (clone SP1 and IE2, respectively). Tumors with ≥ 1% of cells showing positive nuclear staining for the expression of ER and PgR were evaluated as ER/PgR-positive. IHC for Ki67 was performed using a mouse monoclonal anti-human Ki67 antibody (MIB-1, Dako). The labeling index (LI) was assessed as the percentage of tumor cells showing definite nuclear staining among > 1000 invasive tumor cells.

### FISH

FFPE tissue Sects. (5-µm thick) were deparaffinized and digested using standard processing methods for FISH. The PathVysion HER2 DNA Probe Kit (Abbott Molecular/Vysis) was used for the hybridization of tissue sections. Hybridization and counterstaining were performed according to the manufacturer’s instructions. Slides were imaged using an Applied Imaging system running Ariol SL200 (Leica Biosystems, Tokyo, Japan). At least 30 nuclei were evaluated, and the results were interpreted following the 2018 American Society of Clinical Oncology and the College of American Pathologists guidelines [15].

### Statistical analysis

For comparisons of the estimated CN among the IHC score groups, data were analyzed using a Kruskal–Wallis test and Steel–Dwass multiple comparison test. ROC analysis was conducted to evaluate the diagnostic performance of estimated CN for IHC3 + or IHC2 + /FISH( +) outcomes. *P* values of < 0.05 were considered to indicate a significant difference. Statistical analysis was performed using SPSS software (version 19.0 for Windows; IBM Corp., Armonk, NY, USA) and R v3.6.1.

## Supplementary Information

Below is the link to the electronic supplementary material.

Supplementary file1 (JPG 152 KB)

Supplementary file2 (JPG 471 KB)

Supplementary file3 (JPG 316 KB)

Supplementary file6 (JPG 176 KB)

Supplementary file4 Details of the clinical and pathological features of the patients with breast cancer (XLSX 18 KB)

Supplementary file5 Table S2: Details of the clinical and pathological features of the patients with non-breast cancer (XLSX 12 KB)

## Data Availability

The data that support the findings of this study are available from the corresponding author, [author initials], upon reasonable request.

## Abbreviations

DIN: DNA integrity number
ER: Estrogen receptor
ERBB2: Erb-b2 receptor tyrosine kinase 2
FFPE: Formalin-fixed paraffin-embedded
HER2: Human epidermal growth factor receptor 2
IHC: Immunochemistry
PgR: Progesterone receptor
ROC: Receiver operating characteristic
CN: Copy number
NGS: Next-generation sequencing
