# Supplementary figures and images for: Estimating copy number using next-generation sequencing to determine ERBB2 amplification status
Source: Med Oncol. 2021 Mar 12;38(4):36. doi: 10.1007/s12032-021-01482-1 (PMC7954749; doi:10.1007/s12032-021-01482-1)

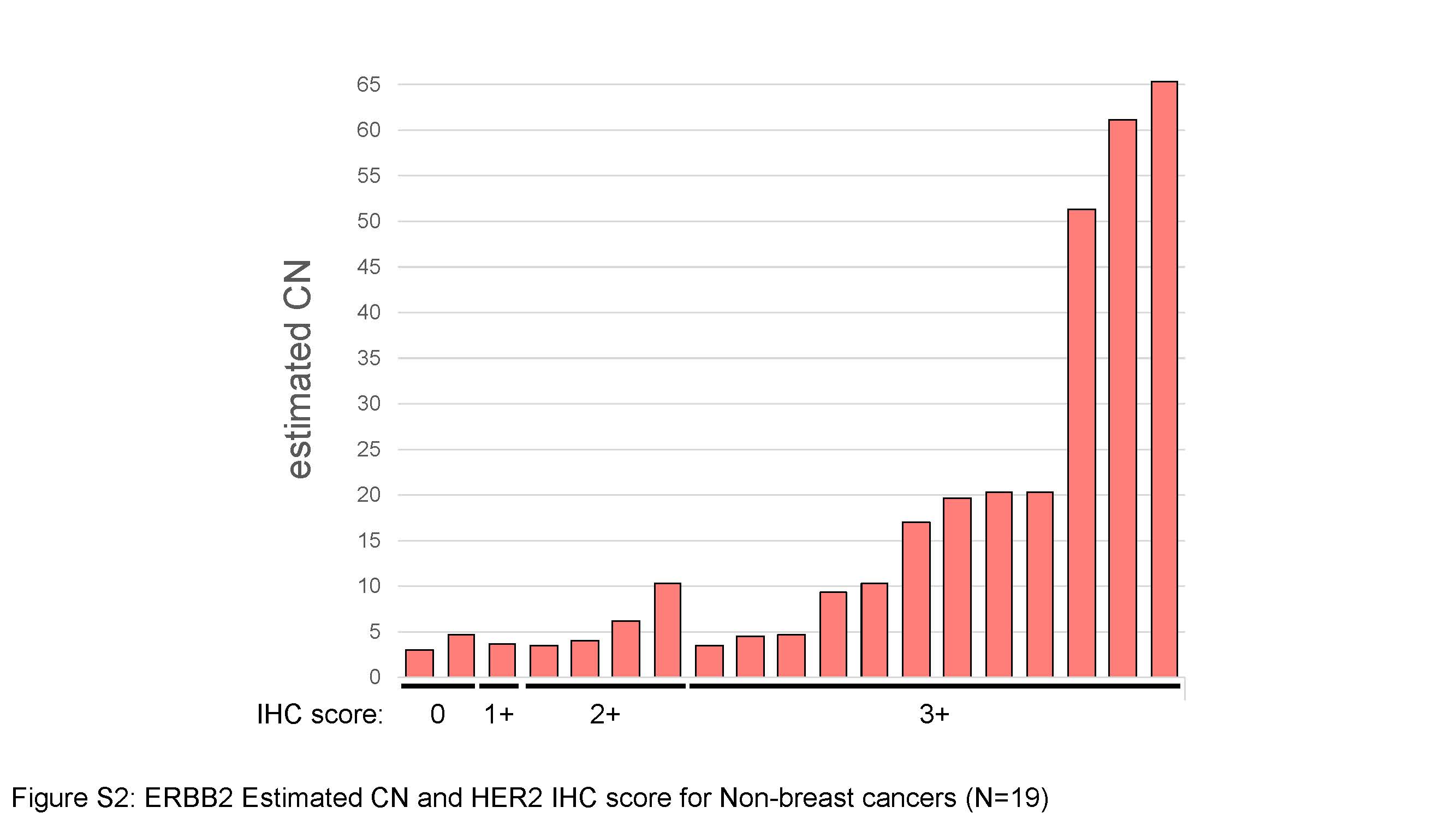

Supplement: Supplementary file 1 — Supplementary file1 (JPG 152 KB) [file 12032_2021_1482_MOESM1_ESM.jpg]

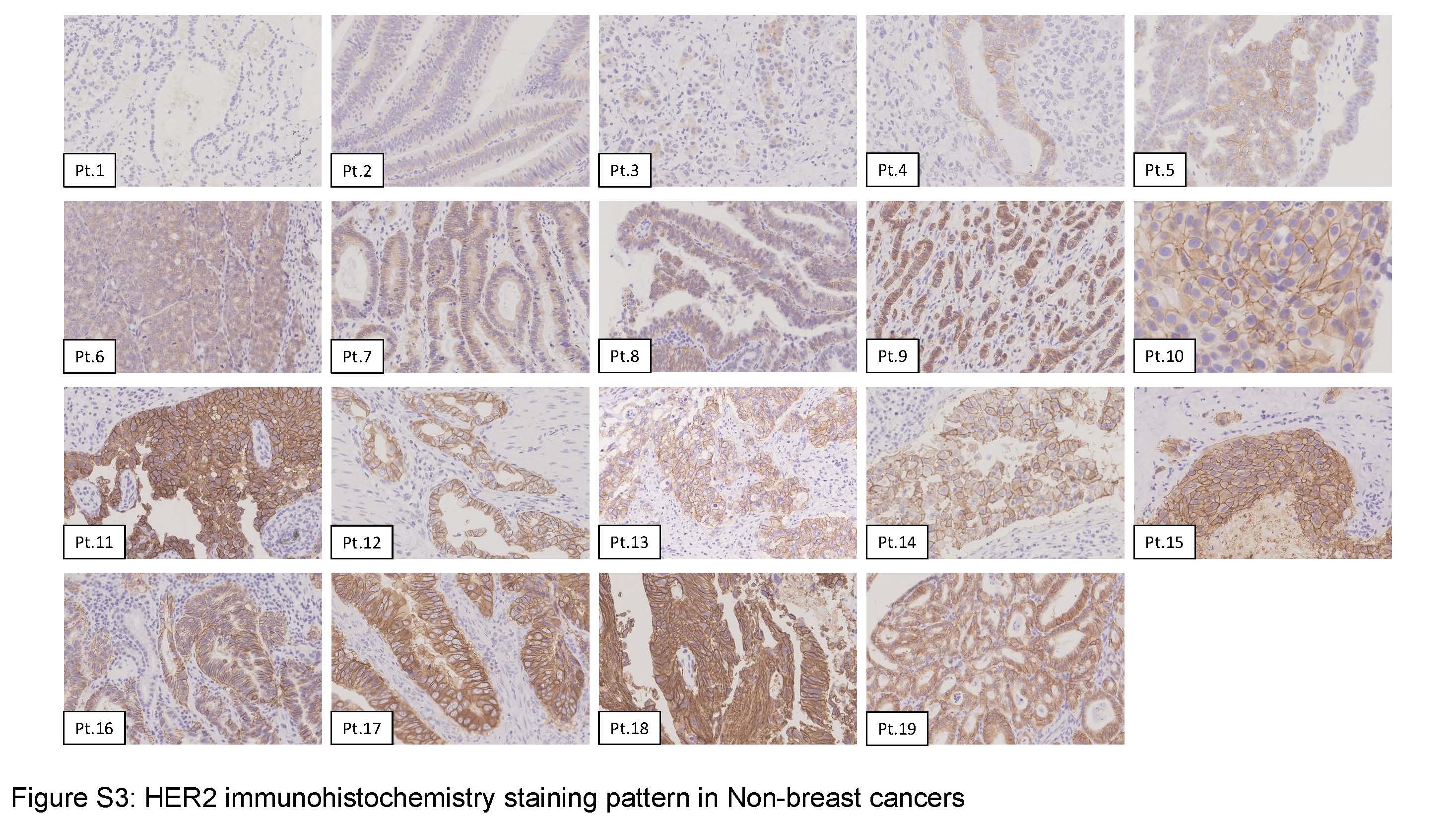

Supplement: Supplementary file 2 — Supplementary file2 (JPG 471 KB) [file 12032_2021_1482_MOESM2_ESM.jpg]

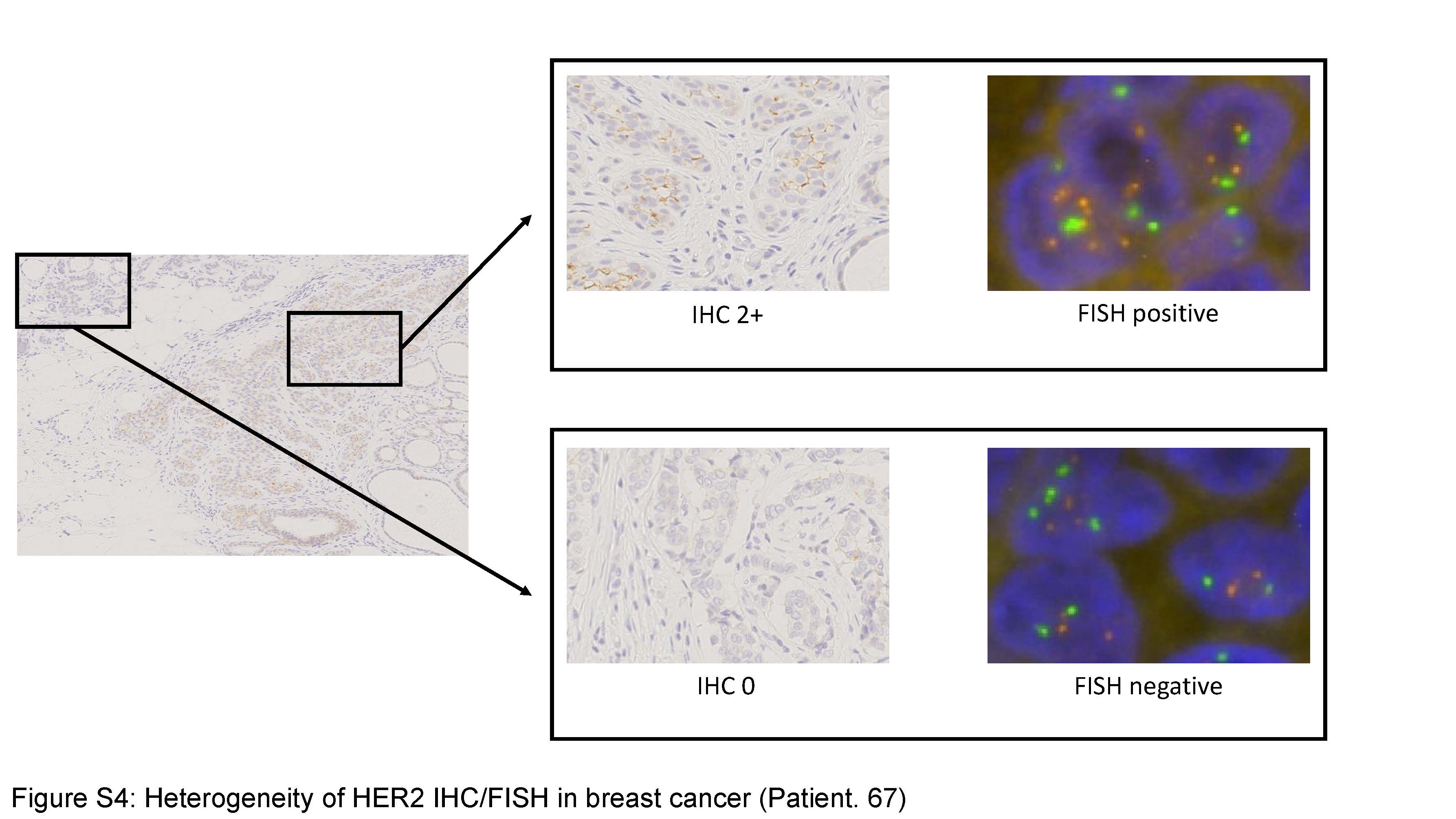

Supplement: Supplementary file 3 — Supplementary file3 (JPG 316 KB) [file 12032_2021_1482_MOESM3_ESM.jpg]

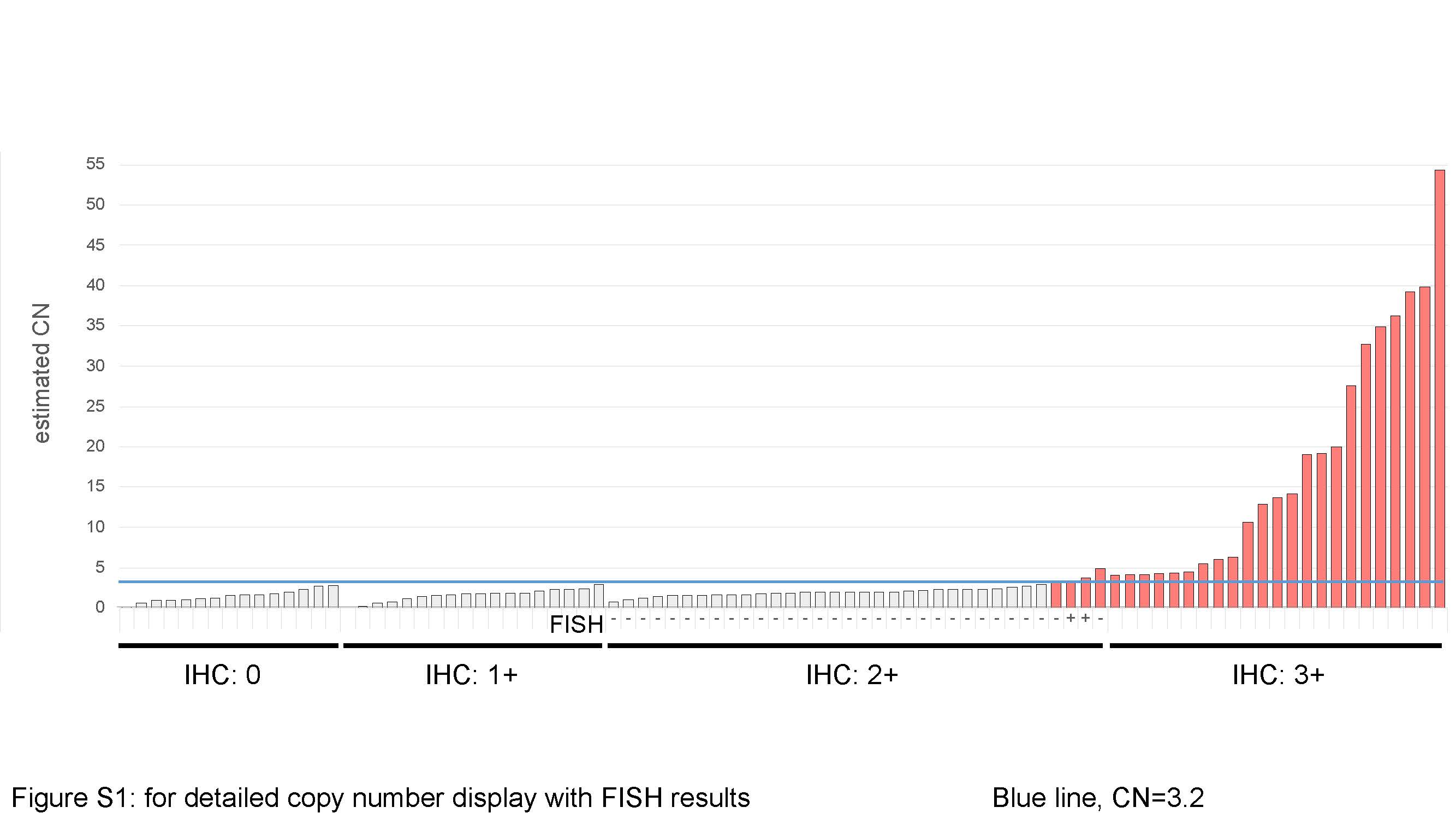

Supplement: Supplementary file 4 — Supplementary file6 (JPG 176 KB) [file 12032_2021_1482_MOESM4_ESM.jpg]
